# A Quantitative 3D Motility Analysis of *Trypanosoma brucei* by Use of Digital In-line Holographic Microscopy

**DOI:** 10.1371/journal.pone.0037296

**Published:** 2012-05-22

**Authors:** Sebastian Weiße, Niko Heddergott, Matthias Heydt, Daniel Pflästerer, Timo Maier, Tamás Haraszti, Michael Grunze, Markus Engstler, Axel Rosenhahn

**Affiliations:** 1 Applied Physical Chemistry, University of Heidelberg, Heidelberg, Germany; 2 Institute for Functional Interfaces, IFG, Karlsruhe Institute of Technology, Karlsruhe, Germany; 3 Department of Cell & Developmental Biology, Biocenter of the University of Würzburg, Würzburg, Germany; 4 Biophysical Chemistry, University of Heidelberg, Heidelberg, Germany; 5 Max Planck Institute for Intelligent Systems, Stuttgart, Germany; University of Texas-Houston Medical School, United States of America

## Abstract

We present a quantitative 3D analysis of the motility of the blood parasite *Trypanosoma brucei*. Digital in-line holographic microscopy has been used to track single cells with high temporal and spatial accuracy to obtain quantitative data on their behavior. Comparing bloodstream form and insect form trypanosomes as well as mutant and wildtype cells under varying external conditions we were able to derive a general two-state-run-and-tumble-model for trypanosome motility. Differences in the motility of distinct strains indicate that adaption of the trypanosomes to their natural environments involves a change in their mode of swimming.

## Introduction

Human african trypanosomiasis (HAT) and nagana are devastating plagues occurring in sub-Saharan Africa with infections of humans and cattle, respectively. Besides their medical importance, these neglected tropical diseases cause enormous economic damage in some 36 African countries [1], [2]. Trypanosomiasis is caused by the unicellular blood parasite *Trypanosoma brucei*, which is transmitted between mammalian hosts by the infamous tsetse fly vector. To date only few drugs are available, which also have severe side effects [1]. The self-propulsion of the unicellular blood parasites is thought to contribute to their virulence. Trypanosomes deploy several ways of evading the host’s immune system. One mechanism is antigenic variation of its surface coat, which is dominated by a single type of variant surface glycoprotein (VSG). A sporadic change of VSG expression allows the cells to escape the humoral immune response. Undoubtedly, antigenic variation is the main mechanism of parasite virulence. A second mechanism has been recently discovered that allows the trypanosomes in an early state of infection to “wash off” surface-bound host antibodies from their cell surface. This process requires cellular motion and rapid endocytosis [3]. The proposed “molecular sails” mechanism exploits the hydrodynamic drag exerted on the surface of moving cells; the shear force specifically pushes antibody-bound VSGs to the posterior end of the cell, where they are internalized via a specialized organelle, the flagellar pocket. The intake rate depends on several factors, amongst them antibody size and speed and directionality of cellular motion. The rate of endocytosis itself is unusually fast but constant.

The propulsion of trypanosomes occurs via beating of a single flagellum attached along the cell body. This motion is driven by a flagellar wave typically propagating in tip-to-base direction during periods of directional swimming. The exact mode of trypanosome motion has intensively been discussed [4], [5], [6] and only recently been deciphered (Heddergott et al., submitted). The mechanism of antibody removal can only be kept up as long as the cell shows directional movement, i.e. swimming with the flagellum leading [3]. Molecular biological studies in combination with light microscopy suggest that trypanosomes do have the ability to reverse the direction of flagellar beating [5], [6], [7], [8], [9]. While pure tumbling has been observed in procyclic (insect form) RNAi mutants [6], swimming, neither of bloodstream form (BSF) nor procyclic form (PCF) trypanosomes had so far been analyzed in three dimensions. This, however is important for quantifying the role of swimming for antibody removal and, hence was one goal of our project. In addition, it is reported in literature [3], [5], [7], [8], [9], [10], [11], that one can paralyze trypanosomes or reverse swimming direction, by means of RNA interference (RNAi) technology [12]. We have exploited this technique to reverse flagellar beat and induce tumbling in BSF trypanosomes.

In this manuscript we apply digital holographic microscopy for a full 3D quantitative analysis of motion patterns of BSF and PCF trypanosomes in varying environments. Classical microscopy motility studies have so far been done on procyclic trypanosomes at room temperature [7], [8], [13], [14]. We have also used the pathologically more relevant bloodstream form trypanosomes under physiological conditions.

## Materials and Methods

### Trypanosome Culture

Wildtype bloodstream form (BSF) *Trypanosoma brucei brucei*, strain 427 [15], [16], Molteno Institute Trypanozoon antigen type 1.6, were cultivated in suspension at 37°C, 5% CO_2_ in HMI-9 medium, including a final volume of 10% FCS (Sigma-Aldrich, Germany). BSF were kept in the exponential growth phase at a cell density below 5×10^5^ cells/ml by dilution with fresh culture medium. Prior to dilution of the cells, the medium was filtered using a 0.22 µm sterile filter to remove particles from the medium, which would lead to reduced holographic imaging quality. PCF strain 29–13 was grown in SDM-79 medium at 27°C without increased CO_2_ to a maximum concentration of 1×10^7^ cells/ml. The cells were harvested right before each experiment by centrifugation (1,400 g for BSF/900 g for PCF, for 10 min at 4°C).

### Holographic Microscopy

The principle of holographic microscopy was introduced by Gabor in 1948 [17]. In digital holographic microscopy, the hologram, a diffraction pattern generated when a sample is illuminated with a coherent, divergent electromagnetic wave, is recorded by CCD- or CMOS-modules. The whole three-dimensional spatial information about the probed object is recorded within a single exposure. Amplitude as well as phase information are preserved since the latter is encoded in the diffraction pattern as a modulation of the amplitude measured by the detector. In this work we apply point source laser in-line holographic microscopy as described earlier [18]. The real space information can be reconstructed from the acquired holograms by applying the Kreuzer implementation of the Kirchhoff-Helmholtz reconstruction formula [18]. A four-dimensional set of information, consisting of three spatial coordinates and a temporal coordinate, is provided if one acquires a sequence of holograms of moving objects such as swimming microorganisms [19], [20], [21], [22], [23]. For this data one can derive characteristic descriptors for motility of a given species in a semi-automatic way involving user intervention [20], [23] or fully automatic as soon as a reliable ground truth has been established for the system under investigation [24], [25]. Holography has successfully been applied in the past to study swimming organisms such as algae, paramecium, dinophyceae and dinoflagellates with high spatial and temporal accuracy [19], [20], [21], [22], [23], [26], [27]. In biology, holographic imaging also found a range of non-tracking applications, such as cell morphometric studies [28]. These examples show that holographic microscopy holds great potential for biological applications.

**Figure 1:**
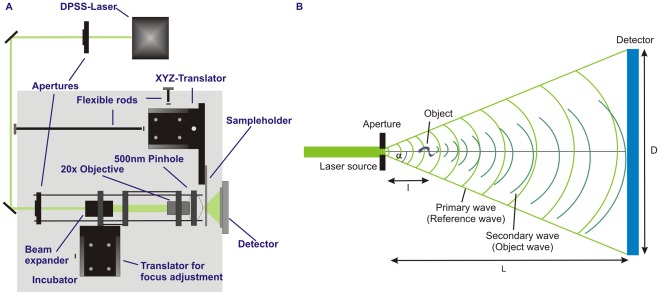
Schematic representation of the digital in-line holographic microscopy setup. A) Schematic drawing of the optical setup used in this study. B) Schematic drawing of the beam path between aperture and detector.

The general design of the digital holographic microscope used in this study follows the idea of using a pinhole to generate a divergent beam for coherent projection microscopy [29]. All optical elements were set up in the in-line geometry. A schematic of the beam path and the setup of the optical elements within the device are given in Figure 1. The light source, a diode-pumped solid-state-laser (IMM Messtechnologie, Germany) working at a wavelength of 532 nm (continuous wave, 30 mW) was used to illuminate a 500 nm pinhole (National Apertures Inc., USA). To improve the photon flux through the pinhole the laser beam was first expanded using a 2x Galilean beam expander (Thorlabs, USA) and then focused by a 20×objective (NA = 0.4, Euromex Microscopes, The Netherlands). A CCD-OEM module (Lumenera Corp., Canada) (1280×1024 active pixels, 8.3×6.6 mm^2^ active pixel area, 8 bit dynamic range, max. frame rate 15.4 Hz) typically run at 10 Hz or a 10 bit dynamic range pco.1200s CMOS-camera (pco.imaging, Germany) (1280×1024 pixels, detector size of 12.3×15.4 mm^2^, max. frame rate 636 Hz) typically run at 5 Hz, were used as detectors. Cameras were positioned at distances of 18–20 mm behind the aperture. In between the pinhole and the camera microcuvettes were positioned which contained the trypanosome suspension. For the used illuminating light cone, distances of 1–2 mm between pinhole and cuvette are a good compromise of magnification and sampling. The detector sizes and distances result in numerical apertures of NA = 0.18 and 0.34, respectively, for the used camera systems. Achievable resolutions in plane range between 1.8 µm and 0.95 µm and in depth from 16.4 µm down to 4.6 µm depending on the camera used, and are thus sufficient to track trypanosomes with a typical size of 3 µm×20 µm. The housing of the device is designed as an incubator with temperature control to suit the demands of the biological samples. Temperatures were stable at 37°C and did not differ by more than 0.1°C between the both sides of the microfluidic system during tests. To keep the device thermally well isolated, positioning of the sample holder was achieved by remotely operating the mechanical translation stages by flexible rods (Haspa GmbH, Germany).

### Sample Cuvettes

**Figure 2:**
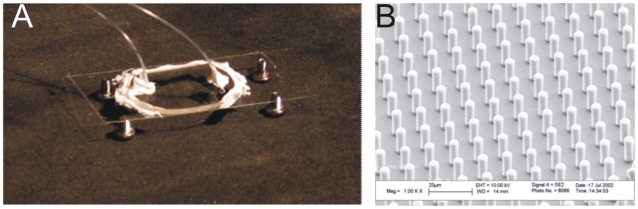
Microstructured sample cuvette used in the study. A) Photograph of pillar channel, B) Electron micrograph of a pillar field with thousand fold magnification.

Experiments were carried out in biocompatible ibidi µ-slide I Luer channels (ibidi GmbH, Germany). The cuvettes used had a length of 5 mm, height of 800 µm and a volume of 200 µl to assure free movement of the trypanosomes within the channel. Experiments to study the motility in a locally confined microstructured array were done in custom-built microfluidic channels. On one lid a pillar array has been created by soft-lithography methods, as described elsewhere [30]. Channels consisted of two parts, 40 µm high channels constructed in a few micrometers thin layer of polydimethylsiloxane (PDMS) on a microscope cover slip. The top part comprised a PDMS block a few millimeters in height structured with a 5×5 mm^2^ pillar field. The pillars were 5 µm in diameter and 15 µm in height, with 20 µm center-to-center spacing [31]. A photograph and an electron micrograph of the pillar array of such a device are depicted in Figure 2.

### Data reconstruction and Analysis

The data presented in this work is based on several datasets typically consisting of 2000–5000 consecutively recorded holograms. Holographic movies have been recorded at 5–10 fps frame rate, and data analysis was carried out with an effective frame rate of 0.5 Hz. This rate was chosen because it corresponds to a travel of about one cell length per frame, giving a sufficient sampling of the cells’ trajectory. Prior to reconstruction, holograms are cropped to their center 1024×1024 pixels. Data reconstruction and coordinate determination for trajectory visualization were carried out according to previously published methods [20], [23], [24], [25]. In brief, after subtraction of a source hologram, single holograms were reconstructed in multiple planes with 5 µm separation. A sufficiently small z-stepping is important to make the depth determination as accurate as possible. The holograms were reconstructed in a 1 mm broad z-range per dataset at varying distances with the minimal distance from the pinhole being 1100 µm and a maximum distance of 2600 µm from the pinhole, depending on the given sample and its position in the beam path. Due to the divergence of the beam [29], the field of view changes with the z-coordinate. For the given datasets the field of view is typically in the range between 200×200 µm^2^ up to 450×450 µm^2^ depending on the distance of the given plane from the pinhole. As described previously by our group, from the volume reconstructions three different projections can be calculated, i.e. the xy-, xz- and yz-projection. From these the centers of mass of the microorganisms’ images are extracted on a frame-by-frame basis by a computer-aided algorithm, which follows single particles and interrupts for user intervention in the case of position uncertainties, e.g. if particles are crossing [23]. The resulting trajectories can be analyzed regarding swimming speeds and angles. Since the setups have relatively small numerical apertures the depth resolution is always worse than the lateral resolution. To take this fact into account, data was smoothed in the z-coordinate in order to avoid that noise alters velocities [24].

## Results

**Figure 3:**
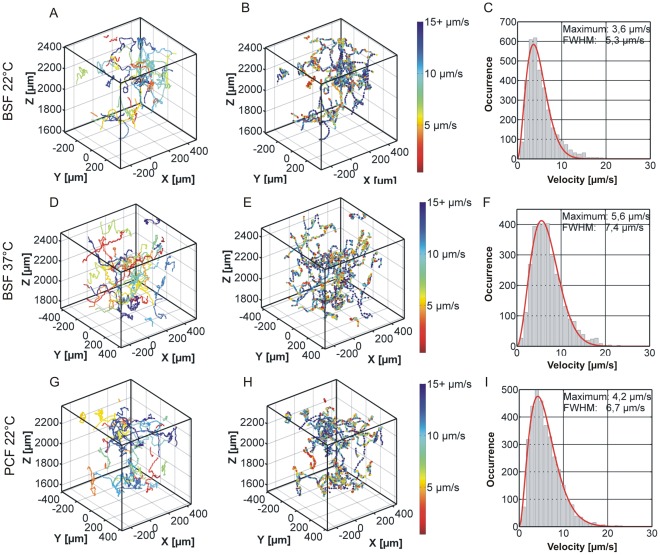
3D representations of trypanosome trajectories. Shown are trajectories of BSF trypanosomes at RT (A) or 37°C (D) and PCF trypanosomes at 22°C (G). In the left column (A, D, G) points with the same color mark traces of the same trypanosome. In the middle column (B, E, H), the color represents the velocity as given by the color bar. Blue points indicate faster movement, red points slower movement. The right column (C, F, I) shows the velocity histograms of the traces.

The goal of this work was to derive a motility model for trypanosomes based on quantitative data and to investigate how the adaption of the trypanosomes either in the bloodstream or the insect form affects motility. One important physicochemical parameter we investigate is the influence of temperature on the motility of the microswimmers. Therefore, we compared the bloodstream forms at physiologic temperature of 37°C and at room temperature. The room temperature data were then compared to the swimming performance of the PCF. It was not possible to analyze recorded procyclic data at elevated temperature since the PCF cells rapidly ceased motion and eventually died during the 37°C measurement most probably due to heat shock. Trajectories as well as an evaluation of the swimming velocities by histograms are shown in Figure 3. At first glance there are no obvious differences in the trajectories. However, the histograms reveal that BSF cells swim slower at room temperature. Interestingly, Figure 3I shows, that the procyclic form swims faster than the bloodstream form at the same temperature. This suggests that swimming velocities of both forms are optimized with respect to their naturally occurring environmental temperature.

**Figure 4:**
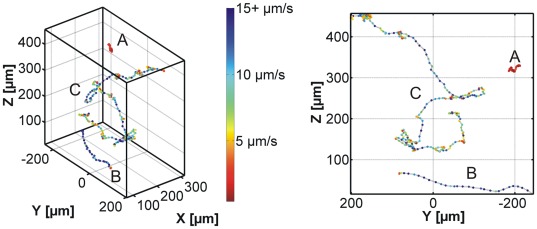
Detectable swimming modes. A) tumbling, B) swimming, C) switching between A) and B).

The trajectories in Figure 3B, E and H are color coded according to the velocity at each given data point. Blue colors resemble faster and red color slower trypanosomes. A closer inspection of the color coded data shows that three different kinds of trajectories can be observed and typical examples for each class are exemplarily shown in Figure 4: cells that swim slow (A), cells that swim fast (B), and cells, which show both, fast and slow motion (C). Class A (slow moving trypanosomes) and class B (fast swimming trypanosomes) can easily be recognized as the velocity and thus color is conserved along the trajectory. In contrast, the behavior of switching cells (C) is characterized by sections of fast swimming, depicted by blue colors, which are interrupted by slow (tumbling) phases, depicted in red colors. Intermediated velocities, indicated by the yellow colors, seldom occur and can mostly be found when a transition between the two swimming modes occurs. We termed the traces in Figure 4 tumbling (A), swimming (B) and switching (C) in analogy to bacterial run and tumble behavior [32].

**Figure 5:**
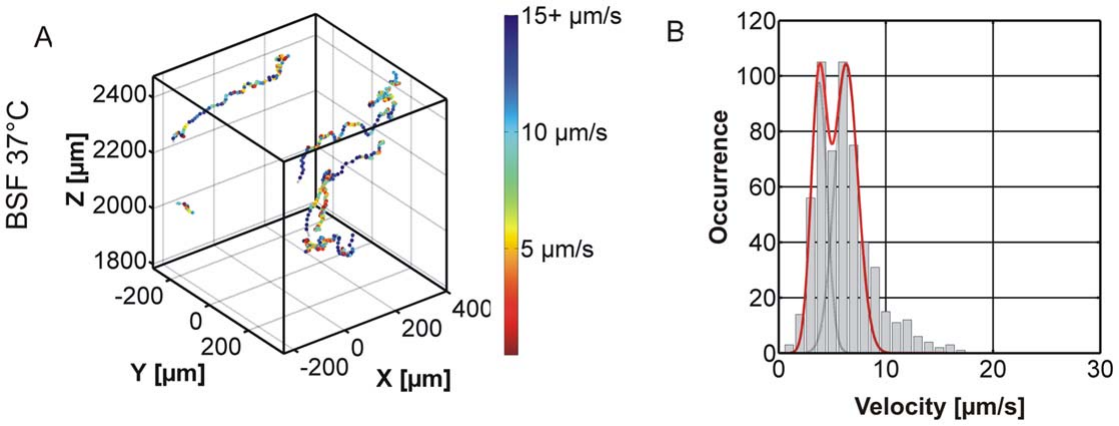
Example for switching trypanosome trajectories from the BSF 37°C dataset. A) shows exemplary trajectories color coded with blue colors indicating fast segments and red colors indicating slower segments. B) Shows the whole class’ velocity distribution with a characteristic dip at 5 µm/s.

A similar, statistical analysis can be done for the switching traces in Figure 5. Switching traces can be found for the PCF, the BSF at 22°C, and the BSF at 37°C. The corresponding velocity distributions in Figure 5B clearly show two maxima with a characteristic dip at velocities around 5 µm/s. This dip will be used below as a threshold to separate switching trajectories into swimming and tumbling phases. The velocities at the two maxima correlate very well with the most probable velocities found for purely swimming (6.4 µm/s versus 7.1 µm/s) and purely tumbling (3.8 µm/s versus 2.7 µm/s) trajectories. The observation of this class of switching trajectories in which two velocities are dominant suggests that trypanosomes have the ability to switch between the two modes tumbling and swimming.

**Figure 6:**
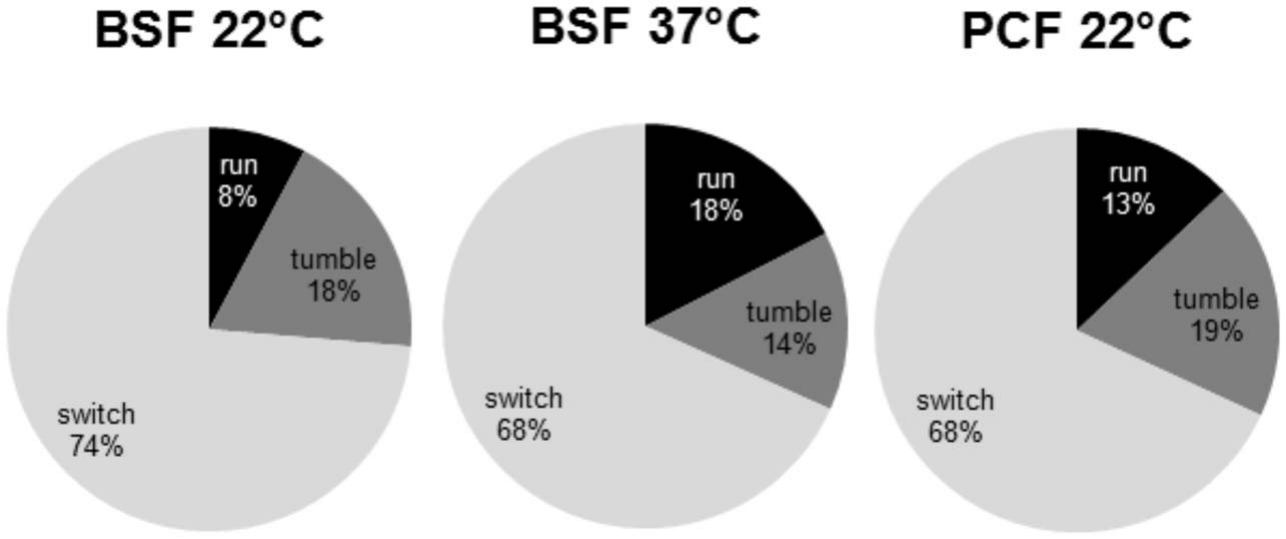
Relative occurrence of the different motion patterns. Shown are relative occurrence of switching (light grey), run (black) and tumble (dark grey) phases for BSF cells at 22°C and 37°C, respectively and PCF at 22°C.

Figure 6 shows the relative occurrence of running, tumbling and switching trajectories. The analysis is based on the assignment of single trajectories with a typical duration of 140 s. The majority of trajectories can be assigned to the class of switchers, while runners and tumblers only play a minor role in their contribution to the ensemble velocity distributions. Already based on the relative occurrence of runners, a clear correlation with the ensemble distributions can be seen where BSF at 22°C reveal the smallest percentage of runners (8%) and the lowest most probable velocity (v = 3.6 µm/s), while PCF trypanosomes show an intermediate behavior (13% swimmers, v = 4.2 µm/s). BSF at 37°C reveal the highest percentage of swimmers (18%) and fastest ensemble speed (v = 5.6 µm/s). In agreement with the general velocity analysis these data support the notion that a decrease of temperature adversely affects motility, which is not unexpected.

As the switching trajectories are the dominant class of patterns, they were analyzed in greater detail. We observe slight differences in the transition between the running and the tumbling phase and only for a minor fraction of the trajectories a clear dip in the velocity distribution as shown in Figure 5 can be observed. This is due to the fact that the switching process between running and tumbling phase can take different times. The longer the transition takes, the more intermediate velocities occur, which broaden the histograms and make it less likely to observe a dip. This becomes obvious when the distributions of all cells (ensemble) are analyzed (Figure 3). No two-peak system can be found and instead the distribution is broad and smears out resulting in one large peak. The velocities in this distribution cover the whole range of swimming and tumbling cells.

**Figure 7:**
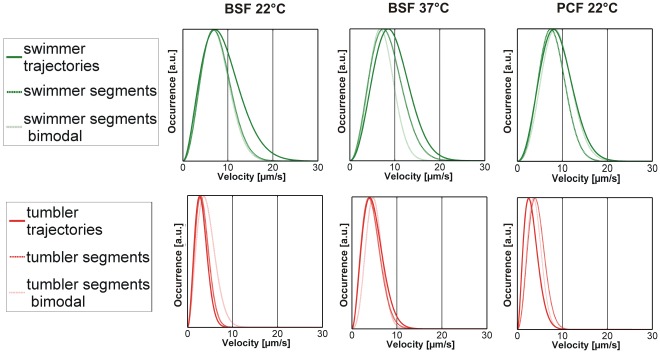
Comparison of the fitted velocity distributions for swimmer and tumbler trajectories. The data for the pure swimmer and tumbler class are compared to the segmented data and to those trajectories which show a clear, “bimodal” switching manifested in the dip in the velocity histograms. The curves have been normalized to their maxima for better comparability.

Visualization of the velocities by the color coding introduced above (blue = fast and red = slow) in Figure 4 and Figure 5 reveals the frequent changes between swimming and tumbling motion in switching traces. As this behavior can easily be determined by eye, we manually segmented the switching trajectories into swimming and tumbling segments using the constraints that a swimming phase must have a velocity >5 µm/s (according to the minimum in the histogram in Figure 5B) and a minimum duration of 6 s. Figure 7 shows the distributions of swimming velocities for the obtained swimmer segments and the obtained tumbler segments. For comparison, the velocity histograms for purely swimming and purely tumbling trajectories are also included. The good agreement of the distributions of swimmer and swimmer segments as well as tumbler and tumbling segments supports the notion that switching traces consist of alternating swimming and tumbling phases. Summarizing, the occurrence of the pure running and tumbling modes and the observation that switching modes have segments of similar velocity (color coded representation), which can result in a bimodal distribution, show that trypanosomes are able to switch between both swimming modes.

### Modeling the Tumbling Phase by Knock-down of a Motility Associated Gene

**Figure 8:**
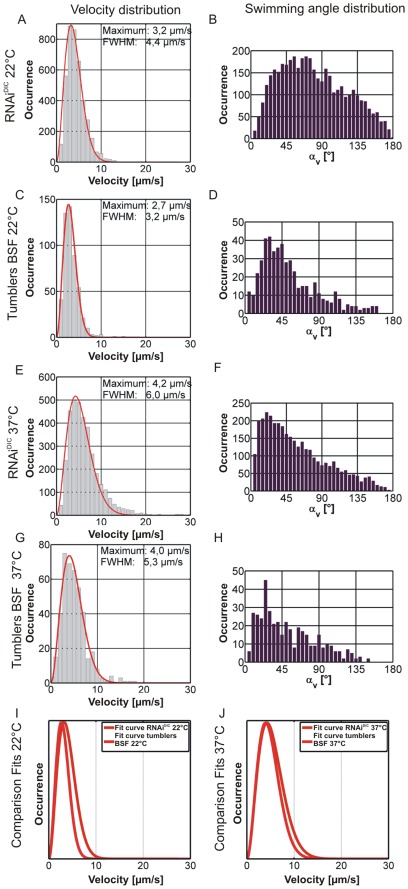
Comparison of swimming parameter distributions for tumbler class trajectories and motility mutant. Shown are the distributions of velocity (A, C, E, G) and swimming angle (B, D, F, H) for tumbler class trajectories of BSF wildtype measurements (C and D for 22°C, G and H for 37°C) and the motility mutant reference dataset (A and B for 22°C, E and F for 37°C) after 12 h of induction of RNA interference against dynein intermediate chain (RNAi^DIC^). Panels I and J show overlays of the fitted curves for the velocity distributions of wildtype tumbler class and motility mutant reference at 22°C (I) and 37°C (J). Curves in I and J were normalized to their maxima.

We investigated a motility mutant (cell line 13–90/pZJM.DIC) with a reversed flagellar beat. Knock-down of a dynein intermediate chain coding gene causes the transgenic trypanosomes to exclusively tumble as shown by Bastin et al. for procyclic trypanosomes after dynein’s light chain knock-down [6]. The distributions of characteristic swimming parameters of the mutant dataset as well as the tumbler traces of BSF wildtype cells at 22°C and 37°C are shown in Figure 8 for comparison. The swimming parameters obtained from the mutant dataset match very well with those acquired for the tumbling trajectories (tumbler class) of the wildtype at both temperatures. The fitted curves of the velocity distributions of wildtype tumblers and mutants show almost perfect agreement (compare Figure 8I and J), which may indicate mechanistic similarity. Both curves resemble tumbler segments (Figure 7). Thus, trypanosomes indeed interrupt their swimming by tumbling phases. Besides the similar motion patterns we can take the data as an indirect hint that the trypanosome tumbling may results from a similar mechanism of propulsion, supporting a model which suggests that the direction of the flagellar bending wave is reversed with respect to the swimming mode (Heddergott et al., submitted).

### Local Confinement as Swimmer Model System

**Figure 9:**
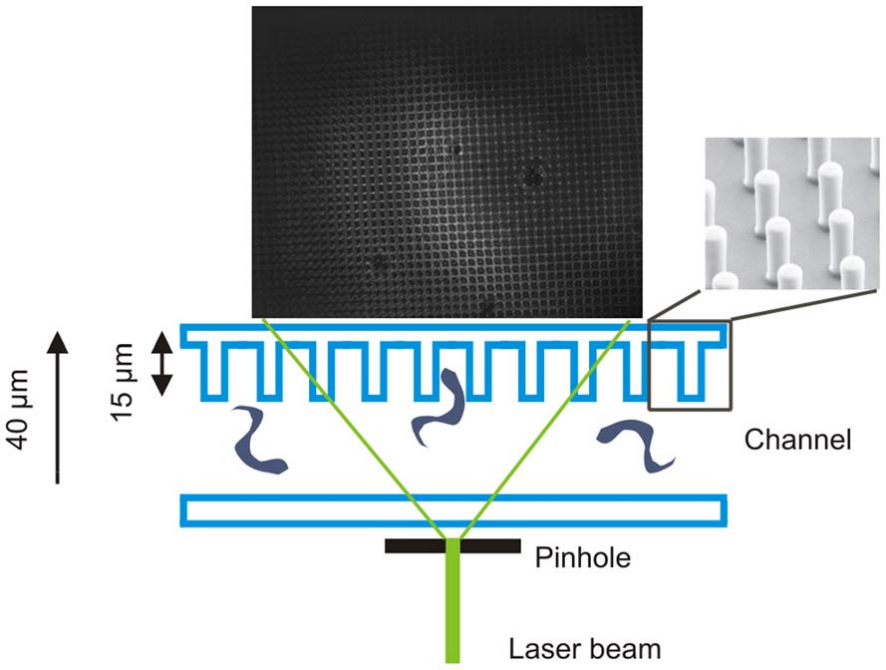
Schematic representation of the configuration of the microstructured channel within the beam path of the holographic microscope.

Since there is no known mutant of *Trypanosoma brucei* in which specifically tumbling is impaired and that always swims directionally, we made use of recent findings revealing that the geometry of the microenvironment greatly influences trypanosome motion behavior (Heddergott et al., submitted). The strategy here was to confine the geometry of the sample cuvette, taking into account that trypanosomes in directional motion show an elongated cell form, while tumbling trypanosomes need more space because they typically exhibit a bent cell shape [33]. The approach was realized by selecting a microenvironment in which obstacles are present that prevent trypanosomes from switching into the tumbling state. The microstructured channels used in this study contained an array of pillars with a spacing of 20 µm (center-to-center) and a height of 15 µm (Figure 2). A schematic drawing depicting the configuration within the beam path of the holographic microscope is given in Figure 9.

**Figure 10:**
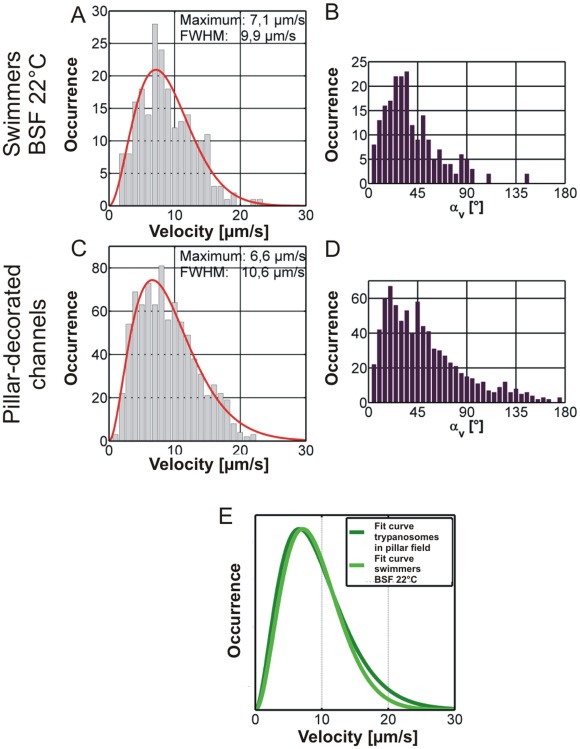
Comparison of swimming parameter distributions for swimmer class trajectories in unstructured channels and trajectories measured in the micropillar array. Shown are the velocity (A and C) and swimming angle (B and D) distributions for swimmer class trajectories of BSF wildtype measurements in structured (C and D) and unstructured (A and B) channels. Panel E shows an overlay of the fitted curves for the velocity distributions of wildtype swimmer class and the ensemble velocity distribution for trypanosomes in pillar-decorated channels at 22°C. Curves in E were normalized to their maxima.

The characteristic swimming parameter distributions acquired from the motility data recorded in the pillar-decorated channel is shown along with the swimmer subclass of bloodstream forms for comparison in Figure 10. A very good agreement for the swimming speed and angle distributions is observed. Especially, the comparison of the fitted curves of the velocity distributions for the compared data (Figure 10E) reveals almost perfect agreement. Unfortunately, it was not possible to measure motion at elevated temperatures as the inhomogeneous heat transfer in the microchannels was inevitably causing convection artifacts. Figure 10E illustrates the remarkable agreement of the swimming model with the purely swimming class of trajectories. Futhermore, the similarity with the swimming segments (Figure 7) reveals that those are mechanistically identical with purely swimming motion (swimming subclass) and the class after stimulation by microstructured arrays.

## Discussion

**Figure 11:**
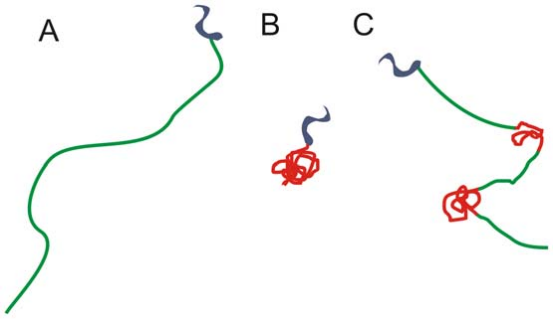
States of the proposed quasi two state system for trypanosome motility. The system consists of swimming (A), tumbling (B) and switching between both states (C). Velocities above 5 µm/s are depicted in green, those below in red.

The detailed analysis of motility data reveals a dip in velocities at 5 µm/s and the comparison with purely swimming, purely tumbling, and segmented trajectories along with two reference systems (genetic knock-down and motility in a microstructured environment) show that trypanosome motility occurs in a two-state-run-and-tumble-mechanism. While microorganisms that switch between both modes are dominant, we also observed few trajectories that for up to 500 seconds revealed either fast and directional swimming (swimmers) or tumbling without relevant net displacement (tumblers). A schematic representation of the observed states is given in Figure 11.

**Figure 12:**
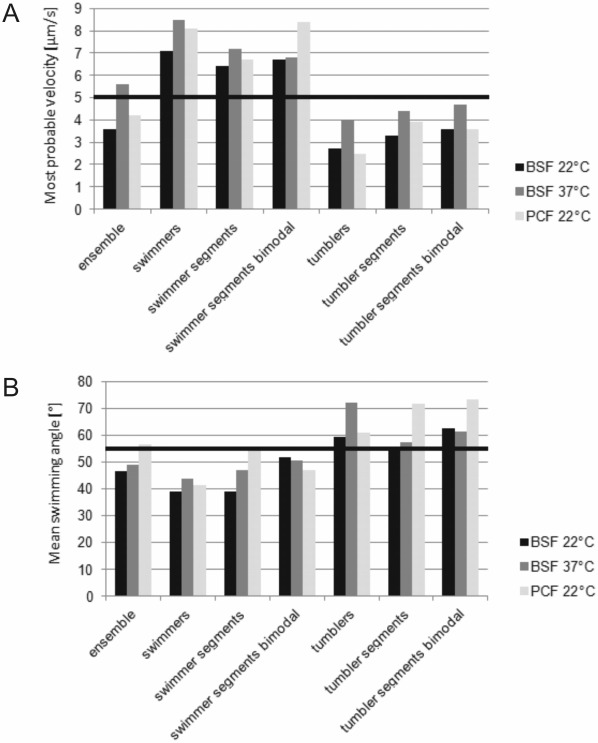
Swimming parameter overview. Depicted is a comparison of (A) most probable swimming velocities and (B) mean angles between two consecutive swimming vectors. The black lines indicate the thresholds between swimming and tumbling at a velocity of 5 µm/s (as derived from the histograms of switching trajectories) and an angle of 55°.

Figure 12 shows a comparison of the most probable velocities and the mean swimming angles for the ensembles, the pure swimmer, the swimming segments, the segments from the bimodal swimmers and the same subclasses for the tumblers at different temperatures. The distributions verify that the threshold for the swimming velocity is well chosen and in good agreement with the segmented data as well as pure swimming and tumbling classes.

**Figure 13:**
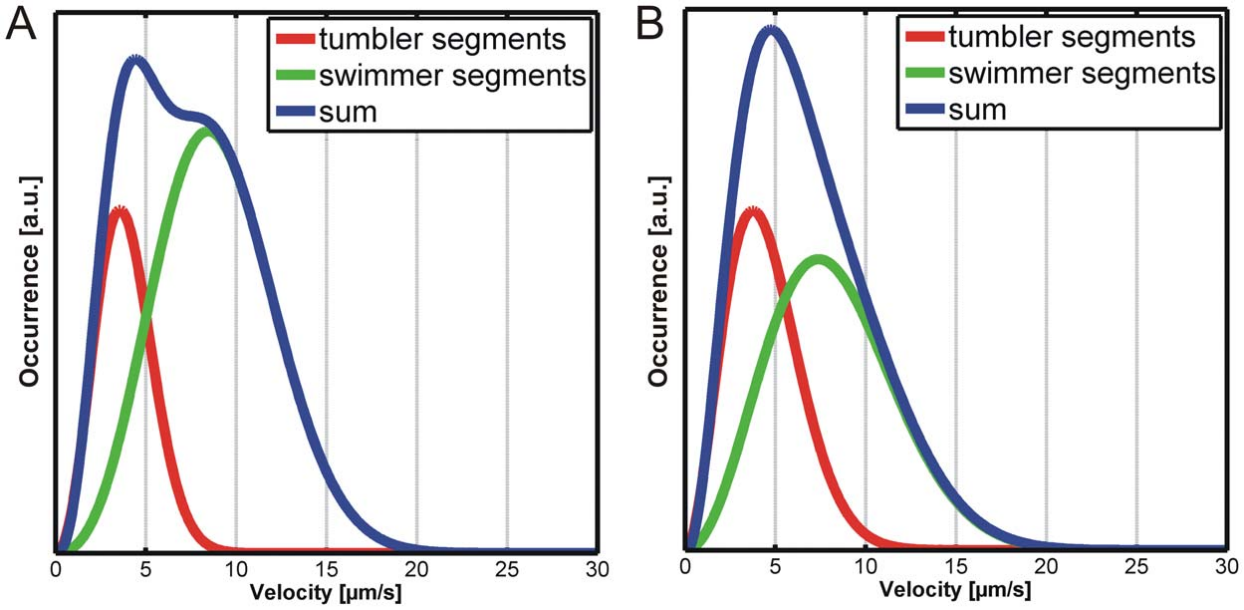
Comparison of bimodal switching and switching behavior without occurrence of a dip in the velocity distribution. Fit curves for bimodal (A) segments and segments of switchers without dip at 5 µm/s (B) and sum curves are shown. Data from PCF dataset.

Based on the extensive analysis of the two swimming modes we can now address the question why in some cases the two behavioral classes can be distinguished (bimodal trajectories), while when considering the full ensemble, the dip in the velocity histogram vanishes and appears as one broad peak. Figure 13 shows the fitted curves over the tumbler (red) and swimmer segments (green) as well as the sum of both (blue) for both cases, bimodal switchers (A) and switchers without dip (B). The visible separation in the summed distribution occurs if (a) the contribution of swimmers to the histogram is large and (b) if the width of the distribution of the two subclasses is small. For all bimodal distributions, the swimming state is well populated and thus, a separation of the two modes becomes recognizable. This observation can be interpreted such that switching becomes immediately obvious if the cells are very motile and prefer swimming instead of tumbling and if the switching phase between the modes is short, which reduces the width of the distribution. Especially the distinctiveness of switching between the modes is affected by all processes related to changes in the underlying molecular machinery. This frequently results in a gradual transition between the two states and thus a broadening of the apparent distributions.

**Figure 14:**
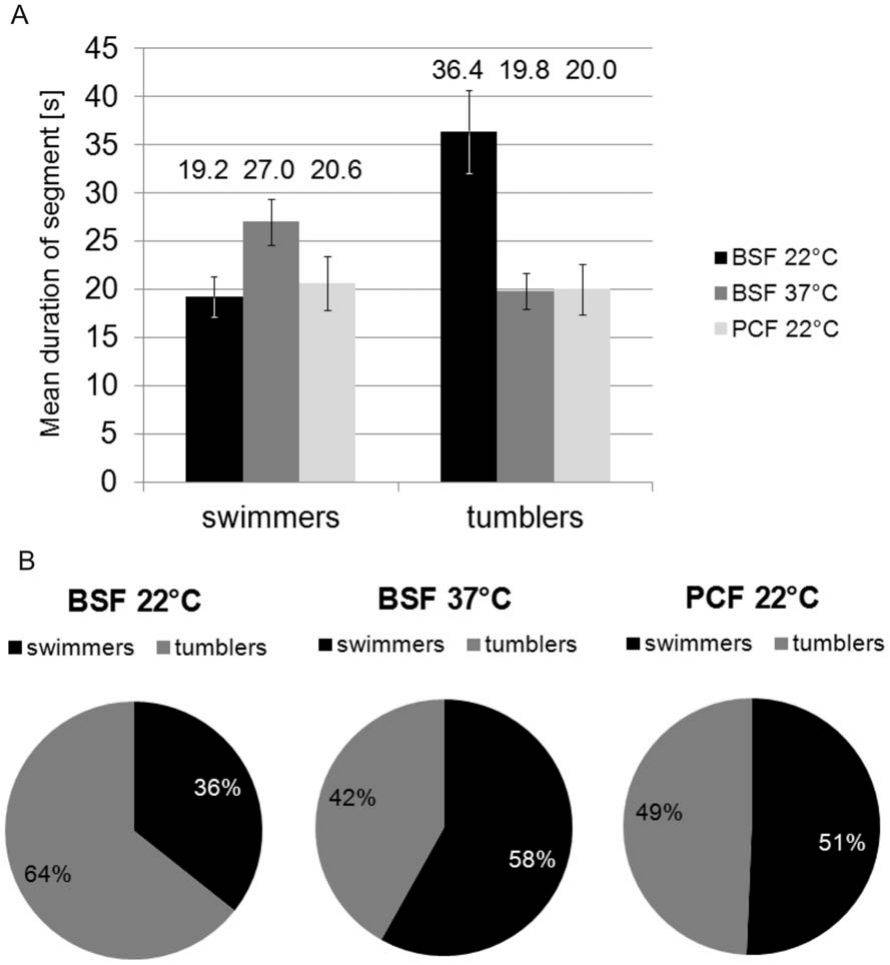
Comparison of switching behavior for BSF and PCF trypanosomes. A) Mean duration of segments for the swimmer and tumbler state B) Distribution of trypanosome swimming vectors contributing to either the swimming or the tumbling state.

In addition to deriving a general motility model for trypanosomes we present evidence for an adaption of the different trypanosome life cycle stages to their natural environments. First hints can directly be derived from the ensemble velocity distributions shown in Figure 3. The bloodstream forms at 22°C show a most probable velocity of 3.6 µm/s, well in the tumbling regime, while at elevated temperature (37°C, physiological conditions) the most probable velocity shifts towards the swimmer regime (5.6 µm/s) or, in other words, the population becomes governed by swimmers. The insect forms show an intermediate behavior. This can be understood by analysis of the temporal switching behavior, i.e. considering the mean segment durations for switchers. The duration of the swimming and tumbling segments as well as the total time spent by the trypanosome in either state is shown in Figure 14. The bloodstream form data clearly indicate more cells swim at 37° when compared to 22°C. Enhanced swimming at elevated temperature is not surprising as most chemical reactions, enzymatic processes, and molecular machinery have activation barriers and are thus temperature dependent. The higher tendency to swim for procyclic trypanosomes at 22°C compared to bloodstream forms at the same temperature can be seen as a sign of adaption of the procyclic cells to lower temperature. This observation seems reasonable as the temperatures in the procyclic cells’ natural environment lie well below 37°C, but they need to swim in order to proliferate within the fly vector.

The two model systems for running (confined micro-geometry) and tumbling (knock-down mutant) are not only important to support the developed run-and-tumble-model but they also reveal important mechanistic implications. The striking similarity of the tumbling mutant with the tumbling segments suggests that flagellar beat reversal plays a major role in the tumbling mode. This connection between swimming mode and direction of the flagellar beat, seen here for bloodstream forms, has so far only been suggested (but not directly shown) for procyclic trypanosomes [6]. Thus, the similarity of tumbling states and mutant data is a strong hint for a mechanistic analogy. In both cases tumbling is induced by flagellar wave reversal, which itself is being reversible in wildtype cells but irreversible in the mutant background.

### Conclusion and Outlook

The swimming behavior of the pathogen *Trypanosoma brucei* was investigated in 3D by digital in-line holographic microscopy. The motility is of general interest as it is a prerequisite for one mechanism of trypanosome immune evasion. We have described in a quantitative manner the swimming trajectories of trypanosomes and have formulated a two-state-model consisting of swimming and tumbling motion as well as switching between both modes. Using segmentation of trajectories, average values for swimming angles and speed have been extracted for wildtype cells and compared to genetically modified trypanosomes, which are only able to tumble. Swimmer traces and segmented swimmer traces agree well with trypanosomes which are stimulated to exclusively swim due to a confinement in a microstructured pillar environment. The data furthermore show that the wildtype life cycle stages of trypanosomes may be adapted to their natural environments also in terms of cell motility. This becomes evident when looking at the behavior of bloodstream cells, which show a clear trend to swim at their physiological temperature of 37°C, while the rate of swimmers and the duration of swimming phases drop significantly when the temperature is lowered. Experiments in microstructured channels confirm that not only elevated temperature, but also introduction of obstacles in the microenvironment triggers swimming. This can be connected with the presence of obstacles, such as red blood cells, in the bloodstream, which are utilized by the trypanosomes to enhance swimming speed. In the future, our insight may be used to correlate the motility of trypanosomes to various other external factors and for screening of new compounds targeted against this deadly disease.
